# The Relationship of Hematological Parameters and C-reactive Protein (CRP) With Disease Presence, Severity, and Response to Systemic Therapy in Patients With Psoriasis

**DOI:** 10.7759/cureus.43790

**Published:** 2023-08-20

**Authors:** Gülsen Şener, Esma İnan Yuksel, Osman Gökdeniz, Kübra Karaman, Harbiye Dilek Canat

**Affiliations:** 1 Biochemistry, Başakşehir Çam and Sakura City Hospital, İstanbul, TUR; 2 Dermatology, Biruni University Hospital, Istanbul, TUR; 3 Dermatology, Başakşehir Çam and Sakura City Hospital, İstanbul, TUR; 4 Biochemistry, Başakşehir Çam and Sakura City Hospital, Istanbul, TUR

**Keywords:** monocyte to lymphocyte ratio, platelet to lymphocyte ratio, neutrophil lymphocyte ratio, crp, psoriasis severity index

## Abstract

Objectives: Systemic inflammation has an important role in psoriasis, which is a chronic disease with an increasing prevalence and is associated with comorbidity. Our aim is to investigate the relationship of hematological parameters and C-reactive protein (CRP) with the presence and severity of the disease in patients with psoriasis. It is also to investigate whether it can be used as a biomarker in monitoring the response to systemic treatment.

Materials and methods: This retrospective study was conducted with the participation of 139 psoriasis patients receiving biological therapy (BT) and conventional therapy (CT) and 140 healthy controls. Demographic, clinical, and laboratory data of patients and controls were examined and all parameters were compared with the psoriasis area severity index (PASI) score. In addition, the changes in these parameters before the treatment and in the third month of the treatment were examined in the patient groups who received BT and CT.

Results: White blood cell (WBC), neutrophil, monocytes, platelet (PLT), plateletcrit, red blood cell, neutrophil-lymphocyte ratio (NLR), and platelet-lymphocyte ratio (PLR) monocyte-lymphocyte ratio (MLR), red cell distribution width (RDW), CRP and erythrocytesedimentation (ESR) levels were higher compared to the healthy control group in psoriasis patients (p<0.05). Baseline PASI values were positively correlated with WBC, neutrophils, monocytes, NLR, MLR, and CRP. WBC, neutrophil, NLR, CRP, and ESR levels decreased in all patients in the third month of treatment (p<0.05). WBC, PLT, neutrophil, and NLR in patients receiving BT; while WBC, neutrophil, NLR, CRP, and ESR levels decreased in patients receiving CT, RDW levels increased (p<0.05). Adalimumab; NLR and basophil, methotrexate; WBC, NLR, neutrophil, and ESR levels caused a significant decrease (p<0.05).

Conclusion: The fact that increased WBC, neutrophils, monocytes, NLR, MLR, and CRP levels are associated with the severity of psoriasis indicates that these parameters reflect systemic inflammation in psoriasis. In addition, the decrease in these parameters after BT and CT suggests that they can be considered simple and reliable markers that can be used as a complement to the PASI score in assessing disease severity and response to treatment.

## Introduction

Psoriasis is a chronic, multisystemic, and common autoimmune inflammatory disease that can occur at any age, affects the skin and/or joints, and negatively affects the quality of life [1]. It is also associated with comorbidities such as diabetes, metabolic syndrome, and cardiovascular disease [2]. Psoriasis vulgaris (PsV), pustular psoriasis (PP), erythrodermic psoriasis (PsE), and arthritic psoriasis (PsA) subtypes have been identified [3]. It is necessary to determine the severity of the disease in order to select the appropriate treatment and to evaluate the effectiveness of treatment [4]. In psoriasis, the psoriasis area severity index (PASI), which assesses the degree of erythema, induration, and desquamation in the affected areas, is one of the most widely used scales to classify disease severity and evaluate treatment [5]. However, due to the fact that PASI measurement is based on subjective criteria and is time-consuming, its use by clinicians is limited, and the search for different scoring systems and markers to measure disease severity continues [4].

Although the cause of this disease, which progresses with remission and relapses, is not known exactly, it is stated that genetic, immunological mechanisms, and subclinical systemic inflammation play an important role in etiopathogenesis [6]. Various inflammatory mediators, including interleukins, chemokines, inflammatory cytokines, and autoantibodies, have been implicated and reported to reflect disease severity [7]. Nevertheless in psoriasis, a generally accepted and definitive reliable biomarker for monitoring disease activity and response to therapy has not been identified. Whereas, since therapeutic management is based on disease severity and affects inflammatory pathways, practical and useful markers are needed to monitor disease progression, provide helpful information to clinicians, and evaluate response to treatment. Parameters such as neutrophil-lymphocyte ratio (NLR), platelet lymphocyte ratio (PLR), and red cell distribution width (RDW), which are relatively stable markers of subclinical inflammation and can be easily measured by complete blood count, have been shown to be useful in the diagnosis and evaluation of some inflammatory diseases [8,9]. Studies investigating C-reactive protein (CRP), NLR, PLR, monocyte-lymphocyte ratio (MLR), and RDW have also been conducted in psoriasis patients [5,9,10]. Nonetheless, as far as we know, no study has been reported that compares all hematological parameters in psoriasis patients with the control group and also compares before and after treatment in patient groups receiving biological therapy (BT) or conventional therapy (CT), including drug subgroups.

The aim of this study is to compare hematological parameters and CRP levels in psoriasis patients with the control group, to evaluate their relationship with PASI score and disease severity. It is also to evaluate the relationship of these parameters with the PASI score in patients treated with different biologics and conventional drugs, and whether they are potential prognostic biomarkers for monitoring response to treatment.

## Materials and methods

This study was approved by the ethics committee of Istanbul Başakşehir Çam and Sakura City Hospital (No:2022.08.261). Due to the retrospective and observational character of the study design, the requirement for informed consent has been waived. This retrospective study was conducted with a total of 139 adult patients with psoriasis who received BT or CT and 140 healthy adults who applied to the Başakşehir Çam and Sakura City Hospital Dermatology outpatient clinic between January 2021 and June 2022. Patients aged 18 years and older who were diagnosed with psoriasis clinically or histopathologically were included in the study. Patients with cardiovascular disease, diabetes, hematological disorder, infection, immunodeficiency, pregnancy, malignancy, and those using drugs that affect platelets in the last two weeks were not included in the study.

Patients with psoriasis were divided into topical BT (26 acitretin, five cyclosporine, 55 methotrexate (MTX)) or CT (six adalimumab (ADA), six ustekinumab, three risankizumab, six guselkumab, six secukinumab, eight ixekizumab) groups according to the treatment they received. Demographic, clinical, and laboratory data of the patients were obtained from patient files and electronic records. The location and type of lesion were recorded. The PASI score was used to evaluate the severity of psoriasis. Psoriasis patients with PASI score < 10 were classified as mild, and those with PASI score ≥10 were classified as moderate and severe. Complete blood count (CBC), CRP, and erythrocyte sedimentation rate (ESR) tests were analyzed in Istanbul Başakşehir Çam and Sakura City Hospital Central Laboratory. CBC was measured with impedance and fluorescent flow cytometry using Sysmex XN-1000 (Sysmex, Kobe, Japan) analyzer. Serum CRP levels were measured quantitatively with the immunoturbidimetric method in Roche Cobas 8000 (Roche, Indianapolis, IN, USA) analyzer, and ESR was measured with the infrared scanning method in Yhlo Vision-C (Shenzhen, China) automatic ESR device.

Descriptive data were expressed as mean ± standard deviation, numerical variables, and percentages. In the analysis of the normally distributed variables, the Student's t-test was applied to examine the differences between the two groups. Differences between two independent groups were examined by using the Mann-Whitney U test for non-parametric variables. The normality of the distribution was analyzed using the Kolmogorov-Smirnov test. The relationship between measurements was analyzed by Pearson or Spearman's Rank correlation analysis and p<0.05 was considered statistically significant. Analyses were performed with SPSS 20.0 software (IBM Corp., Armonk, NY, USA) at a 95% confidence level/interval.

## Results

Demographic and clinical characteristics

This study included 139 (72, 51.8% male, mean age 42.57±14.25; 67, 48.2% female, mean age 42.40±13.56) adult psoriasis patients and 140 healthy controls (70, 50% male, mean age 42.40±14.30; 70, 50% female, mean age 42.04±16.43). The mean disease duration was 11.28±9.51 years in men and 12.25±10.31 years in women. Psoriasis type was PsA in 41 patients (30%), chronic plaque in 123 patients (89%), guttate in 13 patients (9%), flexural in eight patients (6%), erythrodermic in four patients (3%), pustular in 11 patients (8%), and palmoplantar in 15 patients (11%). Nail involvement was present in 90 patients (65%). Thirty-five patients were receiving BT, 86 patients were receiving CT, and 18 patients were receiving topical therapy (Table 1).

**Table 1:** Demographic and clinical characteristics of patients with psoriasis PASI = psoriasis area severity index; PsA = arthritic psoriasis

		Biological therapy n=35	Conventional treatment n=86	Topical treatment n=18	Total n=139
Sex	Female/Male	11/24	46/40	10/8	67/72
Age (years)		46,1±13,0	41,1±14,8	42,1±13,6	42,4±14,6
BMI (kg/m^2^)		28,44±5,83	26,71±5,17	27,36±5,43	26,96±5,40
Psoriasis duration (years)		14,7±10,4	9,8±9,0	15,4±11,0	11,7±9,9
PASI		16,1±9,1	10,0±6,1	10,3±4,6	11,6±7,3
Alcohol consumption		3/35	6/86	3/18	12/139
Cigarette smoking		16/35	34/86	6/18	56
Clinical forms					
PsA		18/35	16/86	7/18	41
Chronic plaque		32/35	76/86	15/18	123
Guttate		2/35	7/86	4/18	13
Flexural		2/35	4/86	2/18	8
Erythrodermic		1/35	1/86	2/18	4
Pustular		0/35	9/86	2/18	11
Palmoplantar		3/35	11/86	1/18	15
Localization					
Scalp		25/35	66/86	14/18	105
Face		11/35	19/86	8/18	38
Body		32/35	81/86	18/18	131
Extremity		33/35	79/86	18/18	130
Palmoplantar		4/35	12/86	2/18	18
Genitalia		15/35	27/86	10/18	52
İntertriginous		3/35	4/86	1/18	8
Nail		28/35	47/86	15/18	90

Laboratory data of psoriasis patients and healthy control group

Psoriasis patients and control group data were analyzed by t-test in independent groups. According to this, WBC, RBC, mean corpuscular volume (MCV), mean corpuscular hemoglobin (MCH), mean corpuscular hemoglobin concentration (MCHC), platelet (PLT), plateletcrit (PCT), neutrophil, lymphocyte, monocytes, basophil, RDW, NLR, MLR, PLR, CRP, and ESR parameters differ significantly between groups (Table 2).

**Table 2:** Laboratory data of psoriasis patients and healthy controls Data are presented as mean ± standard deviation (SD); *p< 0.05 PASI = psoriasis area severity index; WBC = white blood cell; RBC = red blood cell; Hb = haemoglobin; MCV = mean corpuscular volume; MCH = mean corpuscular hemoglobin; MCHC = mean corpuscular hemoglobin concentration; PLT = platelet; MPV = mean platelet volüme; PCT = plateletcrit; PDW = platelet distribution width; RDW = red cell distribution width; NLR = neutrophil to lymphocyte ratio; MLR = monocyte to lymphocyte ratio; PLR = platelet to lymphocyte ratio; C-reactive protein = CRP; ESR = erythrocyte sedimentation rate

	Healthy controls	Psoriasis patients (pre treatment)	p
	Mean ± SD	Mean ± SD	
Sex (Male/Female)	70/70	72/67	0.428
Age (years)	42.22 ± 15.01	42.49 ± 14.3	0.879
BMI (kg/m^2^)	26.56 ± 5.43	27.24 ± 5.39	0.354
PASI basal	-	11.58 ± 7.29	-
Psoriasis duration (years)	-	11.75 ± 9.88	-
WBC, × 10^3^ /µL	7.15 ± 1.41	8.74 ± 2.71	0.000*
RBC, × 10^6^ /µL	4.73 ± 0.32	4.96 ± 0.54	0.000*
Hb, g/dL	13.85 ± 1.06	14.03 ± 1.82	0.319
MCV, fL	87.18 ± 3.28	85.5 ± 4.83	0.001*
MCH, pg	29.16 ± 1.41	28.58 ± 3.04	0.044*
MCHC, g/dL	33.43 ± 1	33.02 ± 1.19	0.002*
PLT, × 10^3^ /µL	260.56 ± 50.16	300.39 ± 76.95	0.000*
MPV, fL	10.53 ± 0.83	10.48 ± 0.86	0.575
PCT, %	0.27 ± 0.05	0.31 ± 0.07	0.000*
PDW, fL	12.41 ± 1.89	12.4 ± 2.08	0.957
Neutrophil, × 10^3^ /µL	3.99 ± 1.01	5.26 ± 2.25	0.000*
Lymphocyte, × 10^3^ /µL	2.36 ± 0.52	2.56 ± 0.84	0.016*
Monocyte, × 10^3^ /µL	0.55 ± 0.15	0.66 ± 0.21	0.000*
Eosinophil, × 10^3^ /µL	0.19 ± 0.12	0.21 ± 0.15	0.248
Basophil, × 10^3^ /µL	0.035 ± 0.017	0.04 ± 0.02	0.002*
RDW, %	12.94 ± 0.76	13.52 ± 1.38	0.000*
NLR	1.76 ± 0.57	2.25 ± 1.12	0.000*
MLR	0.24 ± 0.07	0.27 ± 0.1	0.023*
PLR	115.25 ± 32.38	127.54 ± 47.79	0.013*
CRP, mg/L	1.28 ± 0.98	7.82 ± 13.77	0.000*
ESR, mm/h	6.68 ± 3.88	15.7 ± 16	0.000*

**Figure 1:**
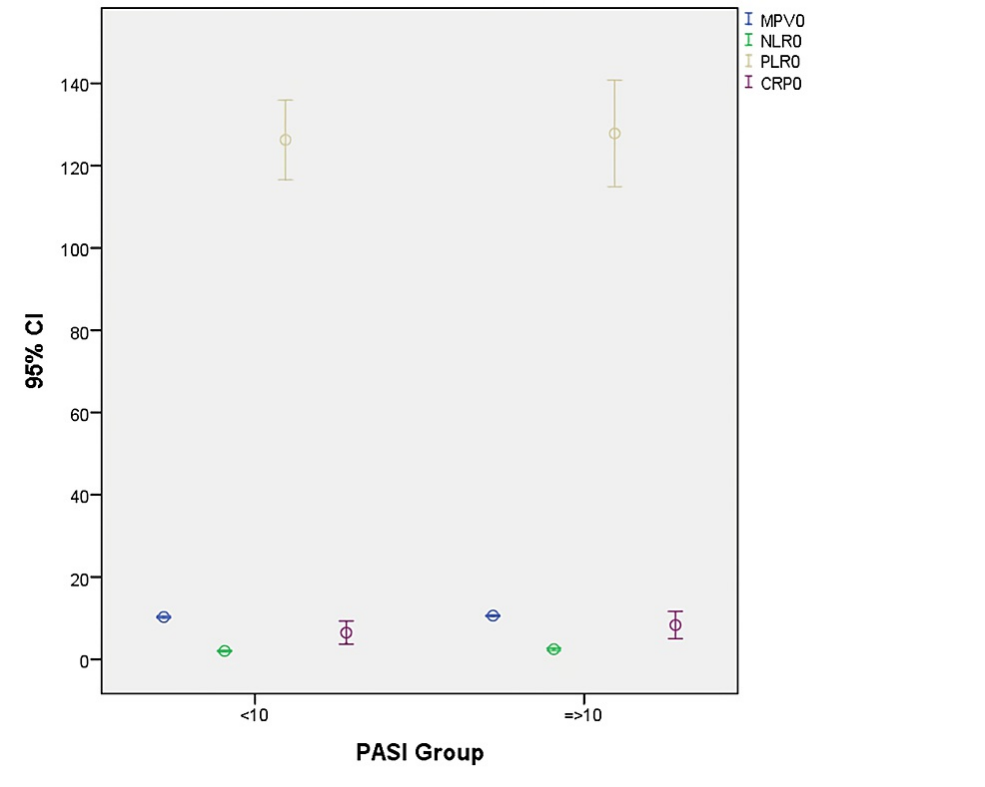
Neutrophil-lymphocyte ratio (NLR), platelet-lymphocyte ratio (PLR), mean platelet volume (MPV) and C-reactive protein (CRP) levels according to basal psoriasis area severity index (PASI) values

Patients were examined by classifying PASI < 10 mild, PASI ≥ 10 moderate to severe according to basal PASI values (Figure 1).

**Figure 2:**
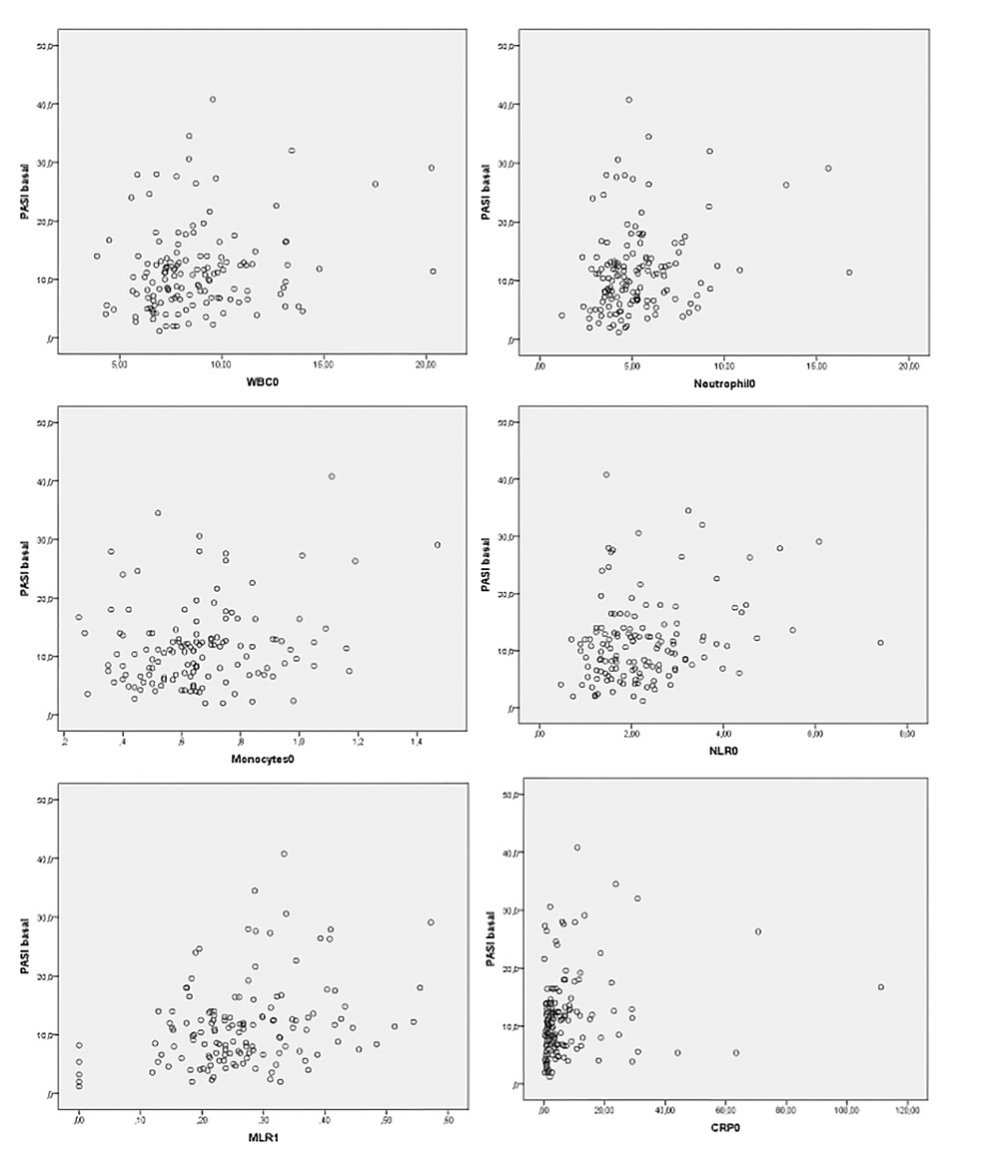
Basal basal psoriasis area severity index (PASI) scores and basal data

Basal PASI score has a positive and significant relationship with WBC, neutrophils, monocytes, NLR, MLR, and CRP (p<0.05) (Table 3, Figure 2).

**Table 3:** The correlation between basal PASI scores and basal data * p< 0.05 PASI = psoriasis area severity index; WBC = white blood cell; RBC = red blood cell; Hb = haemoglobin; MCV = mean corpuscular volume; MCH = mean corpuscular hemoglobin; MCHC = mean corpuscular hemoglobin concentration; PLT = platelet; MPV = mean platelet volüme; PCT = plateletcrit; PDW = platelet distribution width; RDW = red cell distribution width; NLR = neutrophil to lymphocyte ratio; MLR = monocyte to lymphocyte ratio; PLR = platelet to lymphocyte ratio; C-reactive protein = CRP; ESR = erythrocyte sedimentation rate

	r	p
WBC, × 10^3^ /µL	.198	.020^*^
RBC, × 10^6^ /µL	.032	.707
Hb, g/dL	.059	.494
MCV, fL	.127	.147
MCH, pg	.001	.989
MCHC, g/dL	-.053	.551
PLT, × 10^3^ /µL	.020	.821
MPV, fL	.090	.297
PCT, %	.102	.248
PDW, fL	.065	.446
Neutrophil, × 10^3^ /µL	.230	.007^*^
Lymphocyte, × 10^3^ /µL	-.087	.308
Monocyte, × 10^3^ /µL	.219	.012^*^
Eosinophil, × 10^3^ /µL	.012	.893
Basophil, × 10^3^ /µL	.083	.347
RDW, %	.040	.652
NLR	.257	.002^*^
MLR	.326	.000^*^
PLR	.135	.116
CRP, mg/L	.186	.029^*^
ESR, mm/h	.103	.228

Data by treatment groups

According to the treatment received by the patient groups, laboratory data before and after treatment were examined. The group receiving BT; WBC, MCH, PLT, RDW, neutrophil, basophil, NLR, CRP measurements show significant differences after treatment compared to before (p<0.05) (Table 4). WBC, MCH, neutrophil, RDW, NLR, ESR, and CRP in CT areas; WBC, MCHC, neutrophil, basophil, RDW, NLR, CRP, and ESR levels in all treatment groups, differ significantly after treatment compared to pretreatment (p<0.05).

**Table 4:** Comparison of the data of the groups receiving biological, conventional and topical treatment Data are presented as mean ± standard deviation (SD). * p<0.05 WBC = white blood cell; RBC = red blood cell; Hb = haemoglobin; MCV = mean corpuscular volume; MCH = mean corpuscular hemoglobin; MCHC = mean corpuscular hemoglobin concentration; PLT = platelet; MPV = mean platelet volüme; PCT = plateletcrit; PDW = platelet distribution width; RDW = red cell distribution width; NLR = neutrophil to lymphocyte ratio; MLR = monocyte to lymphocyte ratio; PLR = platelet to lymphocyte ratio; C-reactive protein = CRP; ESR = erythrocyte sedimentation rate

	Biological therapy		p	Conventional treatment		p	Topical treatment		p	All treatments		p
	Month 0	Month 3		Month 0	Month 3		Month 0	Month 3		Month 0	Month 3	
WBC	8.92 ± 2.41	8.03 ± 1.69	0.002*	8.69 ± 2.77	8.07 ± 2.01	0.016*	8.64 ± 3.1	8.03 ± 2.48	0.183	8.74 ± 2.71	8.05 ± 1.99	0.000*
RBC	4.92 ± 0.48	4.84 ± 0.54	0.196	4.95 ± 0.55	4.96 ± 0.54	0.868	5.06 ± 0.67	4.93 ± 0.58	0.090	4.96 ± 0.54	4.92 ± 0.54	0.229
Hb	13.99 ± 1.57	13.93 ± 1.81	0.644	13.97 ± 1.76	14.1 ± 1.76	0.123	14.39 ± 2.48	14.2 ± 2.28	0.359	14.03 ± 1.82	14.07 ± 1.83	0.566
MCV	86.43 ± 4.24	86.99 ± 4.03	0.243	85.02 ± 4.43	85.28 ± 4.57	0.306	85.88 ± 7.49	85.83 ± 7.8	0.932	85.48 ± 4.85	85.78 ± 4.95	0.162
MCH	28.42 ± 1.98	33.03 ± 1.1	0.000*	28.69 ± 3.38	33.29 ± 1.06	0.000*	28.34 ± 3.27	33.43 ± 1.43	0.000*	28.58 ± 3.05	28.52 ± 2.03	0.764
MCHC	32.85 ± 1.08	33.03 ± 1.1	0.189	33.1 ± 1.11	33.29 ± 1.06	0.064	32.96 ± 1.76	33.43 ± 1.43	0.064	33.02 ± 1.2	33.24 ± 1.12	0.005*
PLT	311.54 ± 64.36	290.83 ± 54.71	0.019*	302.34 ± 76.68	300.76 ± 74.55	0.725	260.71 ± 90.49	260.71 ± 92.14	1.000	299.53 ± 76.56	293.26 ± 73.14	0.091
MPV	10.53 ± 0.83	10.68 ± 0.62	0.056	10.4 ± 0.86	10.42 ± 0.82	0.804	10.7 ± 0.89	10.9 ± 0.96	0.206	10.48 ± 0.86	10.54 ± 0.81	0.071
PCT	0.32 ± 0.06	0.31 ± 0.06	0.157	0.31 ± 0.07	0.31 ± 0.07	0.720	0.29 ± 0.09	0.28 ± 0.09	0.439	0.31 ± 0.07	0.31 ± 0.07	0.329
PDW	12.62 ± 2.08	12.42 ± 1.42	0.466	12.13 ± 2.01	12 ± 1.84	0.299	13.27 ± 2.22	13.29 ± 2.09	0.959	12.4 ± 2.08	12.27 ± 1.82	0.246
Neutrophil	5.4 ± 2.1	4.64 ± 1.29	0.007*	5.2 ± 2.32	4.64 ± 1.49	0.019*	5.28 ± 2.35	4.87 ± 1.96	0.282	5.26 ± 2.25	4.67 ± 1.5	0.000*
Lymphocyte	2.47 ± 0.59	2.47 ± 0.66	0.977	2.59 ± 0.9	2.53 ± 0.81	0.217	2.57 ± 1	2.32 ± 0.79	0.137	2.56 ± 0.84	2.49 ± 0.77	0.104
Monocyte	0.72 ± 0.21	0.69 ± 0.22	0.281	0.65 ± 0.21	0.63 ± 0.17	0.223	0.62 ± 0.18	0.67 ± 0.3	0.365	0.67 ± 0.21	0.65 ± 0.2	0.251
Eosinophil	0.23 ± 0.14	0.2 ± 0.12	0.196	0.21 ± 0.16	0.22 ± 0.15	0.349	0.14 ± 0.12	0.15 ± 0.14	0.525	0.21 ± 0.15	0.21 ± 0.14	0.810
Basophil	0.05 ± 0.03	0.04 ± 0.02	0.012*	0.04 ± 0.02	0.04 ± 0.02	0.081	0.04 ± 0.02	0.03 ± 0.02	0.126	0.04 2± 0.02	0.036 ± 0.02	0.001*
RDW	13.56 ± 1.42	13.96 ± 1.2	0.010*	13.38 ± 1.15	13.74 ± 1.34	0.003*	13.54 ± 1.14	13.88 ± 1.69	0.227	13.45 ± 1.21	13.81 ± 1.34	0.000*
NLR	2.29 ± 0.95	1.99 ± 0.69	0.041*	2.25 ± 1.24	1.97 ± 0.79	0.024*	2.18 ± 0.84	2.32 ± 1.21	0.608	2.25 ± 1.12	2.02 ± 0.83	0.011*
MLR	0.3 ± 0.09	0.29 ± 0.08	0.349	0.27 ± 0.1	0.26 ± 0.09	0.785	0.24 ± 0.09	0.29 ± 0.1	0.025*	0.27 ± 0.1	0.27 ± 0.09	0.884
PLR	132.82 ± 41.66	122.34 ± 27.03	0.112	128.05 ± 50.65	126.25 ± 38.05	0.623	112.51 ± 45.33	118.62 ± 44.85	0.472	127.34 ± 47.91	124.3 ± 36.34	0.311
CRP	10.05 ± 13.56	6.11 ± 5.59	0.060	7.13 ± 14.87	4.2 ± 8.22	0.047*	6.76 ± 7.17	4.03 ± 3.01	0.093	7.82 ± 13.77	4.66 ± 7.15	0.003*
ESR	18.91 ± 14.03	16.31 ± 9.97	0.148	15.35 ± 17.88	12.15 ± 12.39	0.008*	11.11 ± 6.5	11.22 ± 7.33	0.955	15.7 ± 16	13.08 ± 11.37	0.004*
	Biological therapy		p	Conventional treatment		p	Topical treatment		p	All treatments		p
	Month 0	Month 3		Month 0	Month 3		Month 0	Month 3		Month 0	Month 3	
WBC	8.92 ± 2.41	8.03 ± 1.69	0.002*	8.69 ± 2.77	8.07 ± 2.01	0.016*	8.64 ± 3.1	8.03 ± 2.48	0.183	8.74 ± 2.71	8.05 ± 1.99	0.000*
RBC	4.92 ± 0.48	4.84 ± 0.54	0.196	4.95 ± 0.55	4.96 ± 0.54	0.868	5.06 ± 0.67	4.93 ± 0.58	0.090	4.96 ± 0.54	4.92 ± 0.54	0.229
Hb	13.99 ± 1.57	13.93 ± 1.81	0.644	13.97 ± 1.76	14.1 ± 1.76	0.123	14.39 ± 2.48	14.2 ± 2.28	0.359	14.03 ± 1.82	14.07 ± 1.83	0.566
MCV	86.43 ± 4.24	86.99 ± 4.03	0.243	85.02 ± 4.43	85.28 ± 4.57	0.306	85.88 ± 7.49	85.83 ± 7.8	0.932	85.48 ± 4.85	85.78 ± 4.95	0.162
MCH	28.42 ± 1.98	33.03 ± 1.1	0.000*	28.69 ± 3.38	33.29 ± 1.06	0.000*	28.34 ± 3.27	33.43 ± 1.43	0.000*	28.58 ± 3.05	28.52 ± 2.03	0.764
MCHC	32.85 ± 1.08	33.03 ± 1.1	0.189	33.1 ± 1.11	33.29 ± 1.06	0.064	32.96 ± 1.76	33.43 ± 1.43	0.064	33.02 ± 1.2	33.24 ± 1.12	0.005*
PLT	311.54 ± 64.36	290.83 ± 54.71	0.019*	302.34 ± 76.68	300.76 ± 74.55	0.725	260.71 ± 90.49	260.71 ± 92.14	1.000	299.53 ± 76.56	293.26 ± 73.14	0.091
MPV	10.53 ± 0.83	10.68 ± 0.62	0.056	10.4 ± 0.86	10.42 ± 0.82	0.804	10.7 ± 0.89	10.9 ± 0.96	0.206	10.48 ± 0.86	10.54 ± 0.81	0.071
PCT	0.32 ± 0.06	0.31 ± 0.06	0.157	0.31 ± 0.07	0.31 ± 0.07	0.720	0.29 ± 0.09	0.28 ± 0.09	0.439	0.31 ± 0.07	0.31 ± 0.07	0.329
PDW	12.62 ± 2.08	12.42 ± 1.42	0.466	12.13 ± 2.01	12 ± 1.84	0.299	13.27 ± 2.22	13.29 ± 2.09	0.959	12.4 ± 2.08	12.27 ± 1.82	0.246
Neutrophil	5.4 ± 2.1	4.64 ± 1.29	0.007*	5.2 ± 2.32	4.64 ± 1.49	0.019*	5.28 ± 2.35	4.87 ± 1.96	0.282	5.26 ± 2.25	4.67 ± 1.5	0.000*
Lymphocyte	2.47 ± 0.59	2.47 ± 0.66	0.977	2.59 ± 0.9	2.53 ± 0.81	0.217	2.57 ± 1	2.32 ± 0.79	0.137	2.56 ± 0.84	2.49 ± 0.77	0.104
Monocyte	0.72 ± 0.21	0.69 ± 0.22	0.281	0.65 ± 0.21	0.63 ± 0.17	0.223	0.62 ± 0.18	0.67 ± 0.3	0.365	0.67 ± 0.21	0.65 ± 0.2	0.251
Eosinophil	0.23 ± 0.14	0.2 ± 0.12	0.196	0.21 ± 0.16	0.22 ± 0.15	0.349	0.14 ± 0.12	0.15 ± 0.14	0.525	0.21 ± 0.15	0.21 ± 0.14	0.810
Basophil	0.05 ± 0.03	0.04 ± 0.02	0.012*	0.04 ± 0.02	0.04 ± 0.02	0.081	0.04 ± 0.02	0.03 ± 0.02	0.126	0.04 2± 0.02	0.036 ± 0.02	0.001*
RDW	13.56 ± 1.42	13.96 ± 1.2	0.010*	13.38 ± 1.15	13.74 ± 1.34	0.003*	13.54 ± 1.14	13.88 ± 1.69	0.227	13.45 ± 1.21	13.81 ± 1.34	0.000*
NLR	2.29 ± 0.95	1.99 ± 0.69	0.041*	2.25 ± 1.24	1.97 ± 0.79	0.024*	2.18 ± 0.84	2.32 ± 1.21	0.608	2.25 ± 1.12	2.02 ± 0.83	0.011*
MLR	0.3 ± 0.09	0.29 ± 0.08	0.349	0.27 ± 0.1	0.26 ± 0.09	0.785	0.24 ± 0.09	0.29 ± 0.1	0.025*	0.27 ± 0.1	0.27 ± 0.09	0.884
PLR	132.82 ± 41.66	122.34 ± 27.03	0.112	128.05 ± 50.65	126.25 ± 38.05	0.623	112.51 ± 45.33	118.62 ± 44.85	0.472	127.34 ± 47.91	124.3 ± 36.34	0.311
CRP	10.05 ± 13.56	6.11 ± 5.59	0.060	7.13 ± 14.87	4.2 ± 8.22	0.047*	6.76 ± 7.17	4.03 ± 3.01	0.093	7.82 ± 13.77	4.66 ± 7.15	0.003*
ESR	18.91 ± 14.03	16.31 ± 9.97	0.148	15.35 ± 17.88	12.15 ± 12.39	0.008*	11.11 ± 6.5	11.22 ± 7.33	0.955	15.7 ± 16	13.08 ± 11.37	0.004*

The relationship between PASI and hematological parameters before and after treatment in biological drug users was investigated by correlation analysis. Before the treatment, there was a positive correlation of 25.7% between PASI and NLR, 32.6% between PASI and MLR, and 18.6% between PASI and CRP (p<0.05). However, the PASI score has no significant correlation with PLR, RDW, and MPV measurements. After the treatment, the PASI score is not associated with any parameter (p>0.05) (Table 5).

**Table 5:** The relationship between psoriasis area severity index (PASI) with neutrophil-lymphocyte ratio (NLR), monocyte-lymphocyte ratio (MLR), platelet-lymphocyte ratio (PLR), red cell distribution width (RDW), mean platelet volume (MPV) and C-reactive protein (CRP) before and after treatment in biologic drug users * p< 0.05

	Pre treatment		After treatment	
	r	p	r	p
PASI & NLR	.257	.002^*^	-.227	.203
PASI & MLR	.326	.000^*^	-.267	.139
PASI & PLR	0.135	0.116	-0.078	0.667
PASI & RDW	0.040	0.652	-0.026	0.889
PASI & MPV	0.090	0.297	-0.034	0.851
PASI & CRP	.186	.029^*^	-.009	.960

Changes in basal and third-month serum NLR, MLR, PLR, RDW, MPV, CRP, and PASI scores were evaluated in those receiving BT. There is a significant difference between the first and second measurements of NLR, RDW, and PASI parameters (p<0.05) (Table 6).

**Table 6:** Changes in psoriasis area severity index (PASI), neutrophil-lymphocyte ratio (NLR), monocyte-lymphocyte ratio (MLR), platelet-lymphocyte ratio (PLR), red cell distribution width (RDW), mean platelet volume (MPV) and C-reactive protein (CRP) levels * p< 0.05

		Basal	3 th month	Difference	p
PASI basal	Mean ± SD	16.1 ± 9.1	2.61 ± 3.85	13.16 ± 8.19	0.000*
	Min-max (med)	2-4.80 (13.00)	0-15.70 (0.80)		
NLR	Mean ± SD	2.29 ± 0.95	1.99 ± 0.69	0.301 ± 0.838	0.041*
	Min-max (med)	0.89-4.73 (2.08)	0.77-3.68 (1.89)		
MLR	Mean ± SD	0.3 ± 0.09	0.29 ± 0.08	0.014 ± 0.086	0.349
	Min-max (med)	0.15-0.54 (0.28)	0.13-0.45 (0.29)		
PLR	Mean ± SD	132.82 ± 41.66	122.34 ± 27.03	10.480 ± 37.99	0.112
	Min-max (med)	72.84-266.67 (118.77)	71.58 - 190.11 (114.92)		
RDW	Mean ± SD	13.52 ± 1.39	13.99 ± 1.19	-0.400 ± 0.804	0.010*
	Min-max (med)	12.00 - 18.60 (13.20)	12.30-18.20 (13.80)		
MPV	Mean ± SD	10.53 ± 0.83	10.65 ± 0.63	-0.147 ± 0.433	0.056
	Min-max (med)	9.30 - 12.60 (10.45)	9.60-11.90 (10.70)		
CRP	Mean ± SD	10.05 ± 13.56	6.11 ± 5.59	3.93 ± 11.95	0.060
	Min-max (med)	0.30-70.70 (5.50)	0.30 - 23.70 (5.00)		

The drugs used in conventional treatment were examined separately. MTX led to a significant decrease in WBC, neutrophil, NLR, and ESR levels (p<0.05) (Table 7).

**Table 7:** Variation of inflammatory parameters according to conventional treatment subgroups * p< 0.05 WBC = white blood cell; RBC = red blood cell; Hb = haemoglobin; MCV = mean corpuscular volume; MCH = mean corpuscular hemoglobin; MCHC = mean corpuscular hemoglobin concentration; PLT = platelet; MPV = mean platelet volüme; PCT = plateletcrit; PDW = platelet distribution width; RDW = red cell distribution width; NLR = neutrophil to lymphocyte ratio; MLR = monocyte to lymphocyte ratio; PLR = platelet to lymphocyte ratio; C-reactive protein = CRP; ESR = erythrocyte sedimentation rate

	Acitretin					Methotrexate					Cyclosporine				
	Month 0		Month 3		p	Month 0		Month 3		p	Month 0		Month 3		p
	Mean	SD	Mean	SD		Mean	SD	Mean	SD		Mean	SD	Mean	SD	
WBC	8.93	3.18	8.08	2.53	0.156	8.70	2.67	8.08	1.80	0.033*	7.42	0.91	7.88	1.44	0.538
RBC	4.86	0.52	4.87	0.54	0.871	5.01	0.56	5.01	0.53	0.990	4.84	0.50	4.89	0.68	0.851
Hb	13.87	1.57	13.98	1.78	0.421	14.05	1.87	14.19	1.78	0.220	13.56	1.63	13.72	1.63	0.707
MCV	85.87	4.82	85.70	4.18	0.665	84.61	4.39	85.17	4.74	0.060	85.62	2.63	84.60	4.81	0.527
MCHC	33.24	1.11	33.48	1.12	0.270	33.08	1.16	33.23	1.06	0.259	32.66	0.27	33.28	0.85	0.214
PLT	301.73	75.06	304.69	84.47	0.691	305.35	79.43	297.85	72.69	0.407	273.00	58.86	284.60	71.21	0.414
MPV	10.30	0.98	10.20	0.91	0.663	10.46	0.82	10.50	0.81	0.412	10.38	0.70	10.22	0.71	0.405
PCT	0.30	0.07	0.31	0.07	0.306	0.31	0.07	0.31	0.07	0.783	0.28	0.05	0.29	0.07	0.614
PDW	11.97	2.04	11.60	1.88	0.220	12.22	2.06	12.22	1.84	0.989	11.92	1.47	11.68	1.54	0.514
Neutrophil	5.28	2.84	4.59	1.75	0.221	5.24	2.14	4.68	1.41	0.025*	4.25	0.87	4.60	1.02	0.561
Lymphocyte	2.82	1.08	2.66	1.05	0.152	2.51	0.84	2.48	0.71	0.586	2.32	0.23	2.41	0.32	0.603
Monocyte	0.63	0.24	0.60	0.18	0.603	0.67	0.21	0.64	0.17	0.288	0.62	0.13	0.61	0.13	0.587
Eosinophil	0.16	0.10	0.18	0.10	0.196	0.23	0.18	0.24	0.16	0.609	0.19	0.12	0.21	0.10	0.614
Basophil	0.04	0.01	0.03	0.02	0.205	0.04	0.02	0.04	0.02	0.166	0.04	0.02	0.04	0.03	0.749
RDW	13.19	0.82	13.53	0.97	0.034*	13.68	1.65	13.74	1.48	0.079	13.36	0.68	14.58	1.00	0.114
MCH	29.97	5.00	28.66	1.57	0.190	28.21	2.45	28.31	1.90	0.663	27.98	0.93	28.18	1.92	0.758
NLR	2.16	1.52	1.83	0.72	0.259	2.32	1.14	2.03	0.85	0.037*	1.85	0.41	1.90	0.27	0.822
MLR	0.21	0.11	0.24	0.06	0.457	0.28	0.11	0.27	0.10	0.410	0.27	0.04	0.25	0.05	0.256
PLR	115.96	34.32	123.54	41.02	0.172	134.83	57.88	126.87	38.47	0.182	117.61	20.40	119.58	31.69	0.872
CRP	7.31	13.46	2.37	2.64	0.071	7.42	16.19	5.27	9.98	0.266	3.06	2.73	2.02	1.88	0.085
ESR	13.88	9.88	11.35	7.59	0.097	16.44	21.14	12.80	14.34	0.037*	11.00	9.75	9.20	10.26	0.295

**Figure 3:**
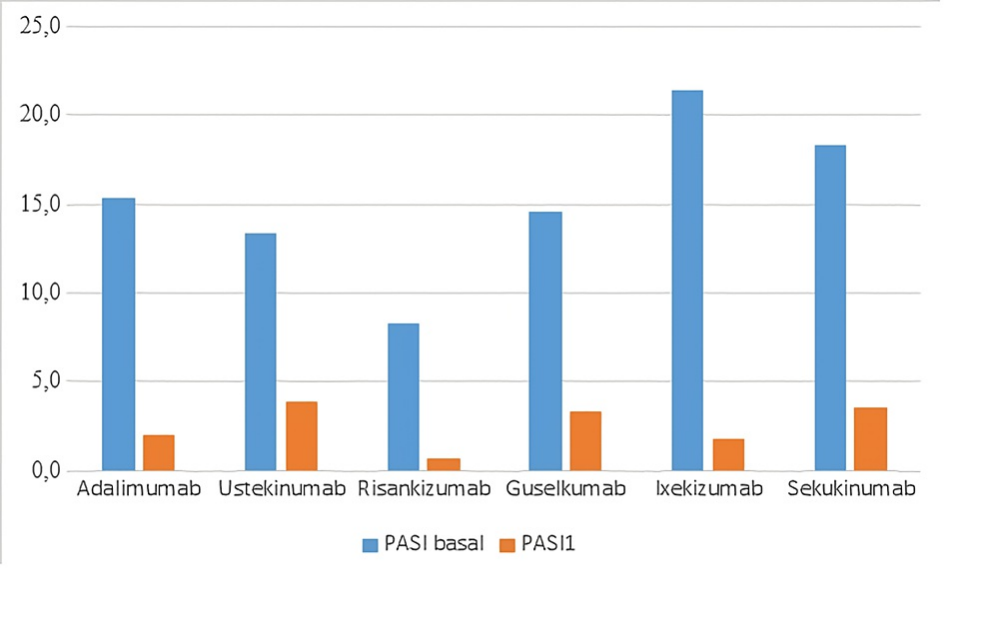
Psoriasis area severity index (PASI) score change according to biological therapy subgroups

The effects of biological drug subgroups on the data were examined one by one. Improvement in PASI scores in treatment with biologic drugs was significant in all subgroups of drugs (p<0.05) (Table 8, Figure 3). (Mean PASI reduction: 91.8% for risankizumab, 91.6% for ixekizumab, 87.2% for ADA, 80.8% for secukinumab, 77.3% for guselkumab, 71.2% for ustekinumab). ADA caused significant changes in NLR, basophil, guselkumab RDW, ixekizumab WBC, secukinumab WBC, and CRP levels (p<0.05).

**Table 8:** Variation of inflammatory parameters according to biological therapy subgroups * p< 0.05 PASI = psoriasis area severity index; WBC = white blood cell; RBC = red blood cell; Hb = haemoglobin; MCV = mean corpuscular volume; MCH = mean corpuscular hemoglobin; MCHC = mean corpuscular hemoglobin concentration; PLT = platelet; MPV = mean platelet volüme; PCT = plateletcrit; PDW = platelet distribution width; RDW = red cell distribution width; NLR = neutrophil to lymphocyte ratio; MLR = monocyte to lymphocyte ratio; PLR = platelet to lymphocyte ratio; C-reactive protein = CRP; ESR = erythrocyte sedimentation rate

	Adalimumab					Ustekinumab					Risankizumab					Guselkumab					İxekizumab					Secukinumab				
	Month 0		Month 3		p	Month 0		Month 3		p	Month 0		Month 3		p	Month 0		Month 3		p	Month 0		Month 3		p	Month 0		Month 3		p
	Mean	SD	Mean	SD		Mean	SD	Mean	SD		Mean	SD	Mean	SD		Mean	SD	Mean	SD		Mean	SD	Mean	SD		Mean	SD	Mean	SD	
PASI	15.30	11.50	1.96	2.24	0.000*	13.33	5.26	3.84	6.78	0.000*	8.20	2.71	0.67	0.70	0.000*	14.47	6.55	3.28	4.46	0.000*	21.36	10.89	1.79	2.58	0.000*	18.25	9.49	3.50	4.26	0.000*
WBC	10.76	4.17	9.02	2.93	0.065	9.84	2.05	9.16	0.82	0.502	9.05	0.53	7.62	1.30	0.305	7.41	1.86	7.81	1.62	0.298	8.34	1.60	7.45	0.85	0.034*	8.38	1.37	7.08	1.15	0.024*
RBC	4.69	0.28	4.65	0.33	0.857	4.91	0.44	4.83	0.18	0.621	5.34	0.07	5.12	0.31	0.417	4.80	0.50	4.57	0.55	0.103	4.93	0.59	4.83	0.57	0.451	5.06	0.58	5.17	0.86	0.568
Hb	13.05	1.15	13.30	1.29	0.562	14.00	1.38	13.77	1.13	0.470	13.70	1.23	13.63	1.55	0.853	14.07	1.56	13.47	2.23	0.169	14.19	1.73	14.11	1.90	0.765	14.73	2.13	15.07	2.41	0.434
MCV	85.44	6.33	87.64	4.04	0.425	87.38	2.84	87.18	3.08	0.730	81.30	3.56	82.80	4.20	0.384	88.82	4.11	89.50	4.40	0.686	86.41	2.97	86.74	3.31	0.529	86.77	4.01	86.62	5.07	0.885
MCHC	32.20	1.14	32.38	1.05	0.472	32.65	0.76	32.63	0.68	0.940	31.50	1.48	32.07	0.81	0.286	32.98	0.86	32.90	1.13	0.656	33.31	1.03	33.68	1.15	0.376	33.47	0.67	33.70	0.97	0.479
PLT	366.50	96.28	315.33	41.73	0.230	301.67	39.92	306.33	31.90	0.588	361.00	14.53	332.00	38.94	0.184	273.50	61.74	265.33	69.52	0.286	301.25	50.20	274.13	58.26	0.163	293.50	48.73	278.00	62.87	0.409
MPV	10.08	0.63	10.38	0.53	0.111	10.58	0.98	10.73	0.92	0.483	10.47	0.93	10.53	0.81	0.529	10.67	0.30	10.80	0.46	0.379	10.53	0.62	10.68	0.49	0.265	10.77	1.41	10.73	0.80	0.905
PCT	0.32	0.07	0.31	0.03	0.890	0.32	0.03	0.33	0.01	0.253	0.38	0.03	0.35	0.03	0.347	0.29	0.07	0.26	0.05	0.235	0.32	0.05	0.31	0.07	0.535	0.34	0.07	0.30	0.07	0.312
PDW	12.17	2.36	11.70	0.85	0.580	12.93	2.69	12.62	2.01	0.684	12.67	1.69	12.43	1.75	0.606	12.37	0.68	12.72	1.36	0.536	12.40	1.25	12.43	1.32	0.963	13.27	3.43	12.63	1.63	0.497
Neutrophil	7.16	3.70	5.22	2.36	0.067	5.90	2.26	4.97	1.07	0.357	5.67	0.56	4.28	1.40	0.233	4.43	1.40	4.77	1.16	0.359	4.77	1.18	4.36	0.71	0.301	4.83	0.90	4.19	0.90	0.122
Lymphocyte	2.47	0.26	2.68	0.65	0.389	2.83	0.53	3.13	0.83	0.216	2.35	0.27	2.35	0.70	0.983	2.18	0.49	2.16	0.52	0.901	2.56	0.72	2.25	0.40	0.148	2.37	0.83	2.28	0.62	0.741
Monocyte	0.81	0.25	0.76	0.25	0.608	0.84	0.20	0.78	0.18	0.416	0.61	0.13	0.67	0.35	0.742	0.56	0.19	0.69	0.30	0.194	0.73	0.21	0.63	0.13	0.134	0.68	0.22	0.62	0.25	0.337
Eosinophil	0.18	0.10	0.13	0.09	0.180	0.21	0.11	0.23	0.13	0.541	0.38	0.15	0.28	0.23	0.344	0.19	0.06	0.19	0.09	0.800	0.23	0.23	0.18	0.14	0.543	0.22	0.11	0.20	0.07	0.673
Basophil	0.05	0.01	0.03	0.01	0.003*	0.06	0.02	0.05	0.03	0.597	0.04	0.02	0.04	0.02	0.802	0.05	0.04	0.04	0.02	0.458	0.04	0.02	0.03	0.01	0.200	0.05	0.04	0.04	0.02	0.363
RDW	13.70	0.77	14.45	0.90	0.138	13.07	1.30	13.53	1.08	0.294	14.60	2.21	14.80	1.37	0.151	12.87	0.46	13.88	0.28	0.009*	13.69	2.04	14.01	1.75	0.085	13.75	0.80	13.80	1.11	0.921
MCH	27.54	2.89	28.38	1.73	0.341	28.55	1.44	28.47	1.54	0.780	25.63	2.31	26.60	1.93	0.413	29.30	1.36	29.44	1.83	1.000	28.80	1.45	29.21	1.32	0.266	29.03	1.54	29.18	1.88	0.704
NLR	2.81	1.14	1.96	0.75	0.044*	2.25	1.17	1.74	0.76	0.283	2.45	0.44	2.00	1.10	0.700	2.05	0.53	2.26	0.54	0.440	2.04	0.89	1.99	0.47	0.891	2.32	1.22	2.00	0.91	0.157
MLR	0.33	0.07	0.31	0.07	0.630	0.31	0.12	0.27	0.09	0.132	0.26	0.03	0.28	0.07	0.617	0.26	0.10	0.34	0.12	0.132	0.30	0.09	0.28	0.05	0.606	0.31	0.13	0.27	0.07	0.346
PLR	148.73	36.84	120.66	15.54	0.153	108.17	13.17	102.63	24.90	0.468	155.10	17.87	147.61	38.54	0.513	131.09	38.77	127.21	34.34	0.676	126.09	43.23	122.08	18.73	0.820	141.12	67.32	126.58	30.64	0.568
CRP	23.68	26.51	6.68	6.94	0.157	9.78	5.93	7.88	5.48	0.489	13.17	15.49	11.10	10.96	0.525	4.43	4.14	5.78	5.54	0.219	8.40	6.73	5.95	2.84	0.189	2.95	2.03	1.83	1.66	0.022*
ESR	28.83	22.44	23.83	14.40	0.231	25.17	19.20	18.33	4.18	0.390	13.33	3.21	15.00	3.46	0.362	15.17	8.86	13.67	9.54	0.580	16.75	6.25	18.25	9.10	0.610	12.17	8.08	7.50	7.58	0.362

## Discussion

Psoriasis is a chronic, multisystemic, and common autoimmune inflammatory disease that can occur at any age, affects the skin and/or joints, and negatively affects the quality of life [1]. Various inflammatory mediators have been implicated in disease formation, but no definitive biomarker has been identified for monitoring disease activity and response to treatment. In our study, we found that WBC, NLR, MLR, PLR, PCT, RDW, CRP, and ESR were significantly increased in psoriasis patients compared to the control group (p<0.05). Basal PASI values were positively correlated with WBC, neutrophil, monocyte, NLR, MLR, and CRP. In patients receiving BT, WBC, PLT, neutrophil, and NLR levels, in CT areas; WBC, neutrophil, NLR, CRP, and ESR levels decreased, and RDW levels increased. The use of ADA caused a decrease in NLR and basophil levels, while the use of MTX caused a significant decrease in WBC, NLR, neutrophil, and ESR levels.

Patients with psoriasis are more likely to have type 2 diabetes mellitus, metabolic syndrome, and increased BMI. It is stated that IL-17 and cytokines released in response have a role in the pathogenesis of diabetes, metabolic syndrome, and obesity [11]. CRP is an acute-phase protein that is mostly released by hepatocytes as a result of the effect of cytokines such as tumor necrosis factor-alpha (TNF-α) and IL-6 [12]. CRP increase contributes to the inflammation cycle by showing a pro-atherogenic effect in psoriasis [13]. In a study, it was shown that the incidence of carotid atherosclerotic plaque in psoriasis patients was higher than in the control group, associated with an increase in inflammatory parameters such as CRP and ESR [14]. It has been stated that CRP elevation, which is considered an independent cardiovascular risk factor, is associated with psoriasis and psoriatic arthritis [11]. CRP levels have also been reported to be a highly reliable systemic inflammatory marker in patients with Japanese psoriasis [15]. Asahina et al., in their study in which they examined 236 psoriasis patients, reported that CRP and NLR were correlated with PASI, as well as elevated CRP and NLR in psoriasis, similar to our results [15]. Therefore, it has been stated that CRP can be used as a systemic inflammation marker in the evaluation of disease severity in psoriasis [16].

It has been stated that the decrease in CRP levels after treatment in psoriatic patients determines the length of remission [16]. Elnabawi's prospective study found that regression in coronary plaque, PASI score, and high-sensitivity CRP (hsCRP) was significant in the psoriatic group receiving BT [17]. In their study, Montaudie et al. showed that the decrease in CRP levels in psoriasis as a result of BT was statistically significant [13]. In some studies, CT has been found to reduce CRP levels [5,18]. In our study, we found that CT caused a statistically significant decrease in CRP levels compared to BT. When we examined the BT subgroups, we found a significant decrease in CRP levels only in patients using the IL-17A inhibitor secukinumab. Similarly, in a study, secukinumab was shown to reduce CRP levels in psoriasis [19]. This condition is explained by the direct antagonistic effect on inflammatory cytokines [13].

Complex relationships between neutrophils, T-lymphocytes, macrophages, mast cells, dendritic cells, and keratinocytes are thought to play a role in the development of psoriasis [12]. Neutrophils increase as a result of an increase in the level of CXCL8, a potent neutrophil chemotactic factor [20]. Neutrophils, which produce various inflammatory mediators, free oxygen radicals, and cytokines, initiate systemic inflammation and demonstrate their active role in the pathogenesis of psoriasis [21]. Rocha-Pereira et al. reported that neutrophil counts increased in psoriasis patients and neutrophil counts were significantly higher in patients with active psoriasis than in inactive patients [22]. Similarly, in our results, it was observed that the neutrophil count was higher in psoriasis patients than in the healthy control group. NLR, which is widely used to evaluate systemic inflammation and severity in bacterial infections and various diseases, is an easily obtained important inflammatory marker and has been found to have prognostic importance [23]. It has been reported that various cytokine levels such as IL-12, IL-6, IL-17, and TNF-α, which are known to increase in psoriasis, cause an increase in NLR levels [21]. Since NLR is also accepted as an independent indicator of cardiovascular disease, it is stated that the increase in NLR in psoriasis patients may be associated with the increase in cardiovascular disease [20]. Therefore, NLR can be used as a potential marker for detecting inflammation in both cardiac and non-cardiac diseases [24]. Paliogiannis et al. stated that NLR adequately reflects systemic inflammation and involvement in patients with psoriasis [25]. In their study, similar to our findings, they showed that NLR values increased significantly in psoriasis patients compared to the control group and were associated with the presence of the disease [25]. Kim et al. also found a positive correlation between NLR and PASI scores, similar to our study, as well as an increased NLR value in psoriasis patients compared to controls [20]. Therefore, NLR is associated with the severity of the disease, as well as psoriasis.

BT has been shown to block neutrophil activation in patients with severe psoriasis [21]. In our study, we observed that BT caused a significant decrease in neutrophil count compared to basal values, without a decrease in lymphocyte count, as reported in previous studies [12,21]. Karagün et al. in their study examining CT subgroups in psoriasis patients, found that MTX was the most effective drug on neutrophils, similar to our results [26]. In a study of 316 patients with psoriasis, Dey et al. showed that increased NLR was associated with PASI and noncalcified coronary artery load, and a decrease in NLR after BT was associated with a change in coronary artery load [27]. In the study conducted by Çevirgen-Cemil et al. it was shown that NLR levels decreased significantly after BT and this situation was independent of the type of biological agent [24]. In our study, we found that NLR and PASI scores could be reduced by BT. Similar to our results, when BT subgroups were examined, a better tendency to decrease NLR levels were detected, especially as a result of treatment with ADA [15]. Karagün et al. in their study, which examined 60 psoriasis patients who received BT and CT, reported that the NLR levels after CT were statistically significantly lower than before the treatment, and it was also found that the NLR and PLR levels in MTX users were statistically significantly lower after the treatment than before the treatment [26]. In our study, too, the decrease in NLR in CT was more significant than in biological agents, and only MTX and ADA caused a significant decrease in NLR levels compared to the subgroups. Therefore, it can be stated that NLR measurement can be used to monitor the severity and course of the disease and the response to treatment, as well as systemic inflammation [12].

Platelets, a rich source of inflammatory cytokines and chemokines, which increase in inflammatory and infective events, are effective on the immune system [12]. PLT plays a role in inflammation in psoriasis by both increasing the migration of leukocytes (WBC) to the skin and binding to endothelial cells [12,15]. PCT, the percentage of PLT in the blood, also predicts platelet aggregation and is used as a cardiovascular disease biomarker [24]. It has been reported that increased polymorphonuclear neutrophil infiltration of PCT in psoriasis is associated with PLT surface antigens, and P-selectin can increase PLT and WBC aggregation in mouse skin by being highly expressed on the PLT surface [28]. As reported in other studies in our study, PLT and PCT, which we found increased compared to controls, support the view that platelets play a role in the pathogenesis of psoriasis [29]. PLR is also associated with chronic inflammatory diseases such as NLR [26]. Similar to our study, some studies have reported that PLR in psoriasis patients shows a significant increase from healthy controls [30]. Activation of PLTs by chronic systemic inflammation in psoriasis has been shown as a possible cause of elevated PLR [25]. Unal et al. stated that PLR is a better inflammatory marker than NLR [30]. Kim et al. reported that NLR and PLR are important inflammatory markers for the presence of PSA [20]. Similar to our study, Wang et al. compared 477 psoriasis patients with 954 controls, did not find a relationship between PLR and PASI despite the significant increase in PLR in psoriasis patients, and stated that PLR may not reflect the severity of the disease while reflecting the inflammatory state [7].

Cemil et al. in a study in which they examined the data of 42 psoriasis patients treated with biologic agents, NLR, platelet, and PCT values were significantly reduced after treatment compared to pretreatment [24]. In our study, we found a significant decrease in platelets, as well as NLR and PLR, which are cardiovascular prognosis markers, in the third month of treatment with biological agents, but the decrease in PCT levels was not significant. This is also consistent with the results of other studies that reported the association of treatment with biological agents with a decrease in major cardiac events observed in psoriasis patients [24]. Asahina et al. reported that NLR and PLR significantly decreased after BT in patients with psoriasis, and this condition is associated with the inhibition of systemic inflammation [15]. It has been stated that PLR levels will be downregulated after BT [15]. Balevi et al. in a study in which 45 psoriasis patients were examined, it was shown that there was no significant difference in terms of NLR and PLR levels between the beginning of the treatment and the third month [9]. Similarly, in our study, regardless of statistical significance, we found a decrease in PLR levels and an improvement in the PASI score, which is defined as treatment success after treatment. However, we found that PLR levels were lower in BT patients than those in CT, although it was not significant, and we think that it can be a guide in monitoring the response to treatment.

Recently, inflammation markers such as NLR, MLR, and PLR have been widely used in the determination of activity and prognosis of some malignant and inflammatory diseases [31]. It has been reported that MLR levels are increased in various systemic autoimmune diseases such as Sjögren's syndrome, rheumatoid arthritis, systemic lupus erythematosus, and ankylosing spondylitis, and there is a positive correlation between acute phase reactants of MLR such as CRP and ESR [32]. Yorulmaz et al. in their study of 171 psoriasis patients, found NLR, MLR, and PLR levels to be increased compared to the healthy control group and suggested that they be used as objective markers reflecting immune inflammatory status in psoriasis patients [31]. Keleşoğlu-Dinçer et al. determined that MLR levels were higher in 106 PsA patients than in 103 healthy controls, and it was reported that PASI correlated with NLR, MLR, and PLR [10]. Similarly, in our study, in addition to the increase in MLR in psoriasis patients, a positive correlation of MLR with PASI was shown.

Although the role of monocytes in psoriasis is not clear, it is known that they cause the secretion of some proinflammatory cytokines. Apheresis removal of increased CD14high and CD16+ monocytes in active psoriasis has been shown to correct the clinical picture of patients with pustular psoriasis and it has been stated that it has an important role [33]. Significant production of TNF-α and interferon gamma (IFN-γ) in CD14high monocytes was detected by Yamanaka et al. and identified as a source of proinflammatory cytokines [34]. It has been shown that biological therapy does not cause any change in monocyte count, but the increased CD14high monocyte ratio in patients with severe psoriasis returns to normal. They stated that they thought that the mechanism of action of biological therapy in psoriasis patients was the result of the inhibition of monocyte activation [34]. In our study, the decrease in monocytes and MLR levels at 12 weeks was not statistically significant in psoriasis patients who received BT and CT.

RDW, which is considered an inflammatory marker, describes the volume and size differences of RBCs. It is stated that an increase in inflammatory cytokines and oxidative stress may cause an increase in RDW [9]. Therefore, it is possible that an increase in RDW in patients with psoriasis indicates an increased inflammatory state [35]. Increased RDW values have been found in rheumatoid arthritis, diabetes mellitus, and Alzheimer’s [9]. Raghavan et al. reported that the RDW level was higher than the healthy controls in their study in which they compared 50 adult psoriasis patients with 50 healthy adults [36]. Coimbra et al. stated that RDW is not a prognostic marker to predict psoriasis severity, but increased RDW is associated with psoriasis vulgaris [37]. In the study of Kim et al. an increase in RDW levels was found in patients with psoriasis compared to the control group, in line with our results [35]. Therefore, RDW is recognized as a potential inflammatory biomarker for psoriasis [28]. It has also been suggested that the increase in the risk of cardiovascular disease in psoriasis patients may be due to the increase in RDW level [24].

Three months of treatment has been found to cause a temporary reduction in RDW levels in psoriasis patients [9]. A study examining the effects of colchicine in patients with recurrent aphthous stomatitis showed that RDW levels decreased [38]. In the study of Balevi et al., which examined psoriasis patients receiving systemic treatment, it was found that RDW levels increased at six, nine, and 12 months, similar to our results. It has been stated that this may be attributed to the transient acute suppressive or destructive effects of the anti-psoriatic drug [9].

Our study has some limitations. In our study, the use of biological and systemic agents was followed for a short time. The number of patients in some treatment subgroups was low. The fact that the study was single-center, the PASI scores of the patients using systemic and topical drugs were not calculated after the treatment may lead to the fact that they cannot fully represent the characteristics of psoriasis patients in our country. Our results should be confirmed by multicenter clinical studies with longer study durations and larger sample sizes. However, our study will contribute to the literature as a study in which all complete blood count parameters in patients with psoriasis are compared with the control group, and also the response to different treatment agents is examined.

## Conclusions

Our results show that hematological parameters may be useful in the early diagnosis of psoriasis and that CRP, WBC, NLR, and MLR can be used as biomarkers positively associated with systemic inflammation in predicting disease severity. WBC, NLR, CRP, and ESR levels with conventional treatment, and WBC, PLT, NLR levels, and PASI scores were significantly reduced with biological treatment. Our data show that MTX is the most effective drug in psoriasis on hematological parameters, that are considered markers of cardiovascular risk. It is extremely important to follow up on the inflammation in psoriasis, which progresses with comorbidity, with inflammation markers. Therefore, we think that complete blood count parameters can be used to monitor disease severity, treatment efficacy, subclinical inflammation after treatment, and develop new therapeutic strategies.
